# Rapid Push vs Pump-Infused Subcutaneous Immunoglobulin Treatment: a Randomized Crossover Study of Quality of Life in Primary Immunodeficiency Patients

**DOI:** 10.1007/s10875-018-0507-x

**Published:** 2018-05-31

**Authors:** Boris Bienvenu, Grégoire Cozon, Yves Mataix, Dominique Lachaud, Antoine Alix, Cyrille Hoarau, Daniel Antier, Eric Hachulla, Sylvie Brice, Jean-François Viallard, Stéphanie Tamisier, Anne-Laure Fauchais, Françoise Renon-Carron, Pierre Clerson, Yann Fardini, Jean-Charles Crave, Pierre Miossec

**Affiliations:** 1 Present Address: Hôpital Côte de Nacre, CHU Caen, Caen, France; 2 Hôpital Saint Joseph, Marseille, France; 3 Centre Hospitalier Edouard Herriot, Lyon, France; 4 Clinique Mutualiste, Lyon, France; 5 Hôpital Bretonneau, CHRU Tours, Tours, France; 6 Hôpital Claude Huriez, CHRU Lille, Lille, France; 7 Hôpital Haut-Lévêque, CHU Bordeaux, Bordeaux, France; 8 Hôpital Dupuytren, CHU Limoges, Limoges, France; 9 Soladis Clinical Studies, Roubaix, France; 10 Octapharma France, Boulogne-Billancourt, France

**Keywords:** Primary immunodeficiency, PID immunoglobulin replacement therapy, home treatment, rapid push

## Abstract

**Purpose:**

Subcutaneous immunoglobulin replacement therapy (IgRT) may be administered once a week with a pump or every other day with a syringe (rapid push). The objective of the study was to compare the impact of pump and rapid push infusions on patient’s life quality index (LQI).

**Methods:**

This study was a randomized, crossover, multicenter, non-inferiority trial conducted in adults with primary immunodeficiency (PID) accustomed to weekly infusions at home by pump. Patients used pump or rapid push for 3 months each according to the randomized sequence. Main criterion was PID-LQI factor I (treatment interference). Non-inferiority ratio was set at 90%.

**Results:**

Thirty patients entered the study; 28 completed the two periods. IgRT exposure was similar during each period. At the end of each period, mean LQI factor 1 was 87.0 (IC95% [80.3; 94.3]) and 77.80 (IC95% [71.5; 84.7]) for pump and rapid push, respectively. There was a slightly larger effect of rapid push on treatment interference than with pump so that the primary endpoint could not be met. No difference was found on other LQI components, satisfaction (TSQM), or quality of life (SF36v2). Eight patients declared to prefer rapid push while 19 others preferred pump. Of rapid push infusions, 67.2% led to local reactions vs 71.8% of pump infusions (*p* = 0.11) illustrating its good tolerance. Rapid push and pump infusions achieved similar trough IgG levels with similar incidence of infections. Rapid push saved 70% of administration cost when compared to pump.

**Conclusions:**

Since IgRT is a lifelong treatment in PID patients, individualization of treatment is of paramount importance. Rapid push is a new administration method in the physician’s armamentarium which is preferred by some patients and is cost-effective.

**ClinicalTrials.gov Identifier:**

NCT02180763

**Clinical Implications:**

Self-administration of small volumes of immunoglobulins at home, every other day, using a syringe (rapid push) is a cost-effective alternative to administration of larger volumes by pump once a week.

**Capsule Summary:**

This study compared subcutaneous infusions of immunoglobulins either weekly via a pump or every other day via a syringe (rapid push). Rapid push is preferred by some patients and is cost-effective, therefore completing a physician’s armamentarium.

## Introduction

More than 50% of primary immunodeficiencies (PIDs) are characterized by an altered antibody production [1], exposing the patients to an increased risk of repeated and severe infections [2]. Immunoglobulin replacement therapy (IgRT) restores sufficiently high serum levels of IgG, decreases the incidence of infections [3], prevents complications such as bronchiectasis, and improves patient’s quality of life [4]. IgRT is administered intravenously (IVIg) or subcutaneously (SCIg). SCIg infusion achieves IgG trough levels and an efficacy similar to those of IVIg infusion but with lower incidence of general reactions [4], better health-related quality of life, higher patient’s satisfaction [5–7], and faster functional recovery with less time off work [8, 9]. Local reactions, however, are more frequent although they tend to decrease with time and patient’s experience [10]. SCIg products are often self-administered at home, therefore modestly disturbing daily activities and reinforcing patient’s autonomy [6, 7]. Usually, patients use an infusion pump and self-administer SCIg once a week. SCIg by pump takes 1 to 2 h even with several catheters infusing several sites all at once.

A new method for SCIg administration has recently been proposed [11, 12]. Rapid and manual administration of SCIg using a syringe, so-called rapid push (RP), would decrease the duration of administration (around 10 min per injection at one or two sites simultaneously) but imposes more frequent infusions. Replacing infusion pumps by RP would also reduce material costs. Those data resulting from retrospective studies urged for a rigorous prospective controlled study to further determine the impact of RP in PID patients. To this end, this randomized crossover multicenter study compared the feasibility, tolerability, and acceptance of RP and the current registered administration in adult PID patients.

## Methods

### Objectives

The main objective was to compare RP and pump administration on interference on daily life in PID patients receiving SCIg. Further objectives included the comparison of other components of specific PID patients’ quality of life, generic quality of life, burden of disease, and of IgRT and patient’s preference for pump or RP. In addition, the study aimed to describe the conditions of infusions and to provide details on costs.

### Study Design

GAMEXPRESS was a prospective, interventional, non-inferiority, longitudinal, randomized, crossover multicenter trial conducted in France in adult patients with PID receiving SCIg at home. Patients had to have a history of ongoing home-based SCIg with gammanorm® 165 mg/mL (Octapharma AG, Lachen, Switzerland) by pump for at least 1 month at the time of enrollment. Eligible patients were randomized (1:1) to pump and then RP or reverse sequence. Each crossover period lasted 3 months. Patients were free to switch from pump to RP and inversely at any time without being dropped out. The dose of immunoglobulins could be adapted during the study as in routine care.

### Immunoglobulins

High viscosity of some IgG solutions can make it difficult to handle the RP plunger by the patient, thus justifying the choice of a low-viscosity product. gammanorm® 165 mg/mL has the lowest viscosity of all SCIg products available and is well tolerated [13]. SCIg was to be administered around once a week during the pump period, and around every other day (three times a week) during the RP period.

Patients were individually trained by the center. The first infusion of each period was performed in a hospital in the presence of an investigator and/or a nurse. Individual phone contacts were set up to accompany the patients during the first week of infusion(s) at home. The proposed maximum infusion rate was set at 1–2 mL/min in order to allow fast diffusion of the product, limit the risk of local untoward reactions, and maintain the patient’s comfort. Patients were allowed to infuse several sites all at once. They were free to premedicate with painkillers, non-steroid anti-inflammatory drugs, or corticosteroids before infusion.

### Evaluations

Patients were evaluated at enrollment (baseline values) and at the end of each 3-month period. At the end of the study, the patients were asked which delivery device they had preferred.

Impact of SCIg infusions on patient’s daily life was evaluated using the PID-specific life quality index (LQI) questionnaire. The PID-LQI questionnaire involves 15 items rated on a 7-point Likert scale ranging from “Extremely Good” to “Extremely Poor.” Since costs for IgRT are entirely covered by the French Social Insurance System, two questions related to economic concerns were deleted. The 13 remaining items are grouped into three subsets measuring the impact of the treatment on patient quality of life: factor I (treatment interference), factor II (therapy-related problems), factor III (therapy settings). Results range from 0 (maximal concern) to 100 (no concern).

Satisfaction regarding SCIg infusion was evaluated by the “Treatment Satisfaction Questionnaire for Medication” (TSQM-11), a generic, self-administered, 11-item scale [14] which evaluates efficacy, ease of use, and overall patient satisfaction. In addition, patients rated their overall satisfaction regarding IgRT at the end of each period, using a 7-point Likert scale. Quality of life was assessed by the SF36v2 scale [15]. The 36 items were grouped into eight dimensions and two summary scores, the physical component summary score and the mental component summary score, were calculated.

PRISM (Pictorial Representation of Illness and Self-Measure) quantitatively assessed the patient’s perception of the burden he/she felt due to his/her illness [16, 17]. This test is based on a non-verbal visualization technique: a rectangular A4-size metal plate represents the life of the patient and a yellow disc located in the lower right corner of the plate represents the patient. The investigator asked the patient to place a red disc on the plate so as to represent the disease in his/her life at the time of the test. The distance between the centers of the discs quantifies the patient’s burden related to the intrusion of the disease in his/her life. The patients were asked to quantify separately the burden of the disease and that of IgRT, and provide additional comments. Since the use of PRISM is not common practice, the investigators were trained prior to study start.

Trough serum IgG levels and serum creatinine were measured at the end of each crossover period. Mild (not interfering with daily activities), moderate (interfering with daily activities), or severe infections (including meningitis, pneumonia, sepsis, osteomyelitis, and visceral abscesses) were reported for each period.

For each infusion, the patients reported the infused dose, the site(s) of infusion, and the number of pumps used (for the pump period), the duration, and the premedication they took before infusion. In parallel, the nurses who followed the patients over the study were asked to report details on the materials that had been used for the infusions.

Adverse events were collected by the investigator at each visit and patients reported local reactions on the diary.

### Statistics

Results were expressed as mean ± standard deviation (m ± SD) or median [first quartile; third quartile] and numbers (percentage). Percentages were calculated on the number of non-missing data. All analyses were performed on an intention-to-treat basis. A sensitivity analysis was performed on a per-protocol subset and provided very similar results (data not shown). Calculation of LQI factors [18], derivation of TSQM dimensions [19], and calculation of standardized dimensions and summary components of the SF36v2 scale [15] were performed as recommended by their authors. Costs were calculated based on public prices, VAT excluded. Costs for nurses were based on the time in hours spent for the infusion multiplied by 29.80 EUR [20]. Time spent for infusion by the patient was valued on the basis of the median income in France (11.68 EUR/h) [21]. For premedication, we calculated a mean price for each type of treatment (for example, the mean cost for painkillers is the mean of public prices for all available painkillers) and costs were calculated by unit. Costs were expressed on a monthly basis (30 days).

Continuous variables were analyzed using a mixed model with delivery device, period, and sequence as fixed factors and patient within sequence as random factor. The effect size associated to delivery device was estimated by the ratio LQI − factor *I*_RP_/LQI − factor *I*_pump_. Results were expressed after exponentiation of the geometric mean of ratios with a two-sided 95% confidence interval (CI). The lower limit of the CI was compared to the non-inferiority threshold of 0.90. Same methods were used for the other variables although not referring to a non-inferiority threshold.

The 3-month incidence of infections was estimated by a Poisson regression model using the natural logarithm of follow-up duration (expressed as a multiple of 3 months) as offset term. The proportion of patients with IgG level < 6 g/L were described for each delivery device along with a two-sided 95% CI (Fisher’s exact method). Proportions of patients rating their satisfaction as rather good or extremely good regarding the P or RP were compared using Fisher’s exact test. Proportions of patients with local reactions, proportions of infusions with local swelling, and proportions of infusions with local pain were compared using two-proportion *z* test.

The analyses were performed using the SAS 9.4 software (SAS Institute, Cary, NC, USA).

## Results

### Patients

**Fig. 1:**
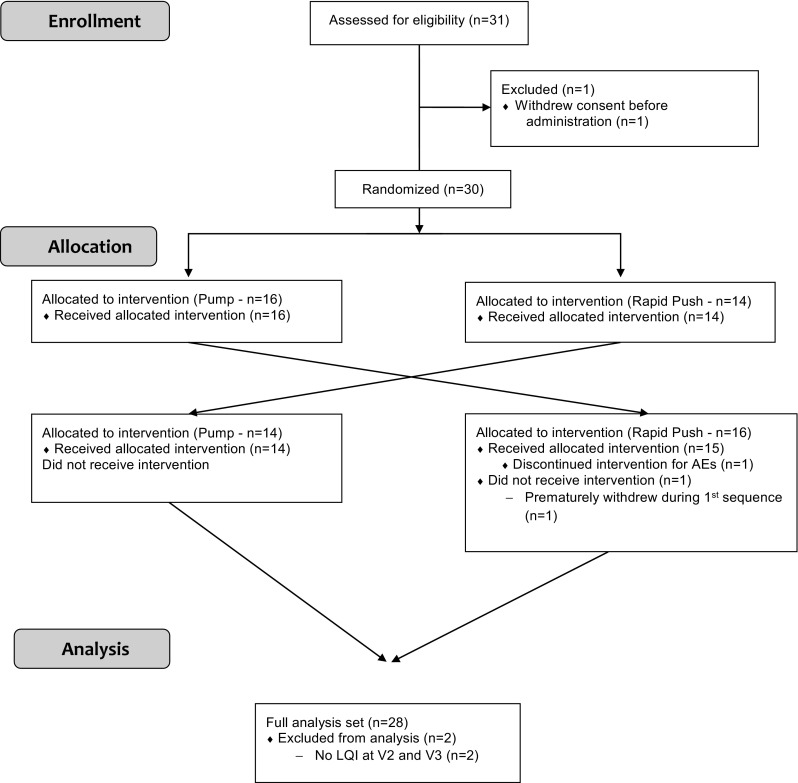
CONSORT flowchart of the study

Thirty patients from six centers entered the study. Two patients who prematurely withdrew for adverse events and did not complete the two periods of the study did not document the LQI scale and were excluded from the intention-to-treat population (Fig. 1).

Patients were aged from 23 to 79 years. All but five were living in couple or family; around half of patients had a professional/school occupation (Table 1). Most frequent PIDs were common variable immunodeficiency (*n* = 17) and hypogammaglobulinemia (*n* = 8). Agammaglobulinemia, severe combined variable immunodeficiency, and selective deficiency of IgG subclasses were less frequent (one patient each). Median time since PID diagnosis was 6.9 [interquartile range 3.8; 22.5] years. Patients had a mean history of ongoing home-based SCIg with gammanorm® 165 mg/mL via an infusion pump of 1.8 ± 2.4 years.

**Table 1:** Characteristics of patients

Number of patients	*N* = 30
Age (years)	49.3 ± 17.8
Male gender	7 (23.3%)
Female gender	23 (73.7%)
Weight (kg)	64.4 ± 16.5
Living alone	5 (16.7%)
Professional/school occupation	14 (46.7%)
Age at PID diagnosis (years)*	37.0 [24.0; 51.0]

Continuous variables are summarized as mean ± SD unless otherwise specified; *median and interquartile range; categorical variables are described as the number of non-missing data and percentage

### Infusions

Patient exposure was similar during pump and RP period. A total dose of 1101.3 ± 569.0 mg/kg was administered with pump and 1101.2 ± 543.3 mg/kg with RP. In total, 355 infusions by pump and 989 infusions by RP have been documented (Table 2). All infusions were self-administered, except the first infusion of each period. Patients received a mean of 12.2 ± 5.0 pump infusions and 38.0 ± 14.7 RP infusions. Mean interval between infusions was 6.6 ± 3.2 days (median 7.0 days) and 2.1 ± 1.1 days (median 2.0 days) with pump and RP, respectively. The total dose administered per infusion was threefold lesser with RP (30.5 ± 14.2 mg/kg) than with pump (94.8 ± 44.3 mg/kg) but patients received the same amount of Ig over each period. Most infusions were done in the abdomen. More than one site was used in 77% of pump infusions but in less than 2% of RP infusions. On abdominal sites, median flow rate was 25 mL/h with pump and 60 mL/h with RP. Smaller volumes and higher flow rates resulted in quicker infusions with RP (range 2 to 70 min, mean 14.1 ± 7.9 min; median 10.0 min) than those with pump (up to 180 min; mean 81.3 ± 44.6 min; median 75.0 min). Premedication with painkillers was reported in not more than 10% of infusions whatever the delivery device was. More specifically, seven patients took at least one painkiller prior to infusion during the RP period. Two patients accounted for 63 out of 86 (73.2%) infusions with RP associated with prior painkiller intake. Five patients took at least one painkiller prior to infusion with P and two patients accounted for 23 out of 36 (63.9%) infusions with P. One patient took painkillers for 100% of infusions with P and 97.1% infusions with RP and counted for 12 intakes during P period and 33 intakes during RP period.

**Table 2:** Characteristics of infusions

	Infusions with pump *N* = 355	Infusions with rapid push *N* = 989
Duration (min)	81.3 ± 44.6	14.1 ± 7.9
Dose administered per infusion (mg/kg)	94.8 ± 44.3	30.5 ± 14.2
Abdomen	310 (87.3%)	802 (81.1%)
Thigh	23 (6.5%)	80 (8.1%)
Other sites	1 (3.1%)	42 (4.2%)
Unknown site	21	65
One site/2 sites/3 sites/4 sites/unknown	74/202/24/21/34	908/16/0/0/65
Volume (mL)*	32.6 ± 15.7 40.0 [30.0; 50.0]	11.2 ± 4.4 10.0 [10.0;10.0]
Flow rate in abdomen (mL/h)	30.9 ± 20.9	54.0 ± 23.3
Flow rate in abdomen [0–15 mL/h]	33 (15.1%)	2 (0.3%)
Flow rate in abdomen [15–25 mL/h]	85 (39.0%)	35 (4.9%)
Flow rate in abdomen [25–35 mL/h]	32 (14.7%)	93 (13.1%)
Flow rate in abdomen [> 35 mL/h]	68 (31.2%)	581 (81.7%)
Flow rate in abdomen unknown	92 + 23 + 11	91 + 5 + 42
Time to perform infusions (min)	81.3 ± 44.6	14.1 ± 7.9
Premedication with painkillers	36 (10.1%)	86 (8.7%)
Premedication with NSAIDs	14 (3.9%)	10 (1.0%)

Continuous variables are summarized as mean ± SD unless otherwise specified; *median and interquartile range; categorical variables are described as the number of non-missing data and percentage; *NSAIDs*, non-steroid anti-inflammatory drugs

### Patient Satisfaction and Quality of Life

**Fig. 2:**
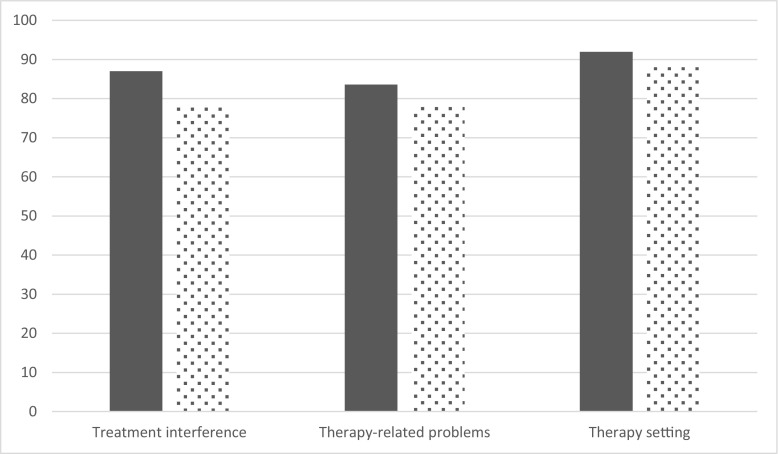
Life quality index (PID-LQI). Legend: dark bars, pump; clear bars, syringe. Values are Lsmeans derived from the mixed model with device, period and sequence as fixed factors, and patient within sequence as random factor

**Fig. 3:**
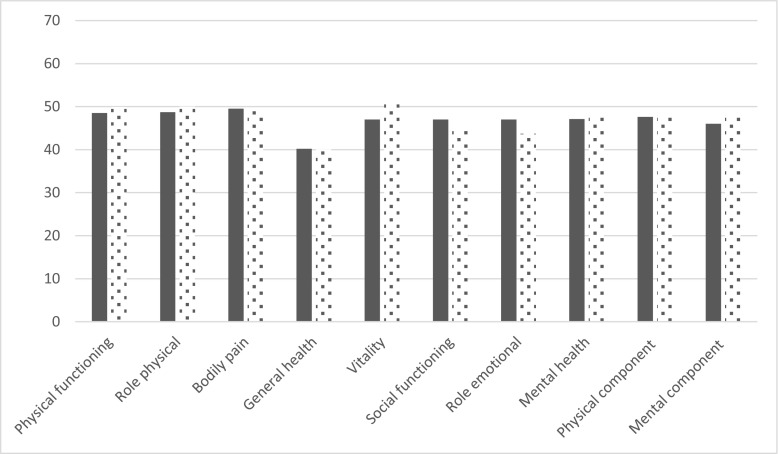
SF36v2 health domain scales and norm-based component scores. Legend: dark bars, pump; clear bars, syringe. Values are Lsmeans derived from the mixed model with device, period and sequence as fixed factors, and patient within sequence as random factor

The LQI factor I (treatment interference) was very high at baseline reflecting that daily life was only slightly altered by home-based SCIg infusion by pump (Table 3). At the end of each period, mean LQI factor 1 was 87.0 (IC95% [80.3; 94.3]) and 77.80 (IC95% [71.5; 84.7]) for pump and RP, respectively. Ratio of least square means (Lsmeans) was 89.4% [80.9%; 99.9%], therefore under the non-inferiority threshold of 90% (Fig. 2). LQI factors II and III were not impacted by the delivery device. At enrollment, TSQM was 73.1 ± 15.4 and satisfaction decreased during the study with poorer results during the RP period than those during the pump period. However, the difference between devices did not reach statistical significance. Quality of life was high at baseline. Apart from general health, other dimensions of SF36v2 were only slightly lower than those in the general population (norm-based values for healthy subjects are 50 for each dimension). The vitality dimension was higher during the RP period whereas no difference was found on other dimensions (Fig. 3). No difference on burden of the disease or burden of IgRT was found between devices. Since comments from patients were sometimes not in line with the PRISM score, additional analyses were conducted on the comments. In total, 27 patients gave a comment for the pump. Among them, 14 were rather positive, 6 were rather negative, and 7 were neutral. For the RP, of 25 statements, 8 were positive, 15 were negative, and 2 were neutral. As a whole, negative statements regarding RP were related to the higher frequency of injections while some patients reported they had difficulties in pushing the plunger. On a 7-point Likert scale, overall satisfaction was rated rather good, good, or extremely good by 26 patients out of 28 (92.9%) when using pump, and by 15 patients out of 28 when using RP (53.6%, *p* = 0.002). Despite this, eight patients declared that they preferred RP (19 preferred pump and one patient did not answer). Patients who preferred RP did not differ from those who preferred pump regarding age, gender, occupation, or duration of IgRT before entry into the study. However, they were more often living alone (4 of 8 who preferred RP versus 1 of 19 respectively who preferred pump, *p* = 0.02).

**Table 3:** Patient’s satisfaction and quality of life

	Baseline *N* = 28	Pump *N* = 28	Rapid push *N* = 28	Ratio* N = 28
LQI factor I*	83.5 ± 12.4	87.0 [80.3; 94.3]	77.8 [71.5; 84.7]	89.4 [80.9; 99.9]
LQI factor II	86.1 ± 12.9	83.6 [77.8; 89.8]	78.5 [72.8;84.7]	94.0 [86.6; 102.1]
LQI factor III	94.1 ± 7.9	92.0 [87.3; 97.0]	88.0 [83.2; 93.1]	95.7 [88.6; 103.2]
TSQM	73.1 ± 15.4	61.8 [49.6; 77.1]	53.4 [42.4; 67.2]	86.3 [64.2; 116.0]
SF36v2: Physical functioning	50.0 ± 8.1	48.5 [44.5; 52.8]	49.9 [45.8; 54.4]	102.9 [98.1; 107.9]
SF36v2: Role physical	47.1 ± 12.1	48.7 [44.6; 53.2]	50.0 [45.8; 54.7]	102.8 [98.0; 107.9]
SF36v2: Bodily pain	49.6 ± 10.0	49.5 [45.5; 53.9]	49.0 [44.9; 53.5]	99.0 [93.2; 105.2]
SF36v2: General health	40.3 ± 11.5	40.2 [36.2; 44.7]	40.1 [36.0; 44.7]	99.7 [92.7; 107.3]
SF36v2: Vitality	49.1 ± 11.0	47.0 [43.0; 51.5]	50.9 [46.3; 55.8]	108.2 [100.6; 116.3]
SF36v2: Social functioning	46.1 ± 11.6	47.0 [42.6; 51.8]	44.4 [40.1; 49.1]	94.3 [86.4; 103.0]
SF36v2: Role emotional	45.9 ± 14.1	47.0 [40.9; 54.0]	43.7 [37.8; 50.5]	93.0 [81.0; 106.7]
SF36v2: Mental health	48.9 ± 11.8	47.1 [42.6; 52.0]	47.6 [42.9; 52.9]	101.2 [93.5; 109.6]
SF36v2: Physical component summary	47.5 ± 7.9	47.6 [44.2; 51.3]	48.0 [44.5; 51.7]	100.7 [97.5; 104.1]
SF36: Mental component summary	46.7 ± 12.9	46.0 [41.1; 51.4]	45.7 [40.7; 51.3]	99.3 [90.5; 109.0]
PRISM–Burden of disease	12.0 ± 7.5	9.5 [6.6; 13.7]	9.5 [6.6; 13.7]	100.7 [79.9; 127.1]
PRISM–Burden of delivery device	10.1 ± 5.6	10.2 [7.9; 13.3]	7.9 [6.1; 10.4]	77.5 [53.3; 112.5]

At baseline results are summarized as mean ± SD; at the end of each period, results are Lsmeans [95%CI]; ratios are calculated as Lsmeans syringe/Lsmeans pump. *For LQI factor I, the non-inferiority threshold was set at 90.0%

### Trough IgG Levels and Incidence Rate of Infections

At the end of each period, patients achieved similar trough IgG levels (9.4 ± 2.3 g/L). Of note, 7 (16.7%) and 5 (11.9%) patients had an IgG level below 6 g/L at the end of the pump or RP period, respectively. Twenty-six infections were reported by 14 patients during the pump period, 25 of which were of mild intensity and 1 was moderate. During the RP period, 19 infections were recorded in 9 patients, 15 of which were mild, 3 were moderate, and 1 was of severe intensity (pneumonia). Six patients had infections during both periods. The overall 3-month incidence rate of infections was 1.00 [0.68; 1.47] for the pump period and 0.76 [0.49; 1.20] for the RP period. Eight patients during the pump period and 6 patients during the RP period (out of 28 patients) received antibiotics at least once.

### Costs

Total direct costs were 1681 ± 628 EUR for pump and 1320 ± 702 EUR for RP. After excluding costs related to gammanorm® 165 mg/mL, monthly direct costs for administration were 536 ± 176 EUR for pump and 164 ± 323 EUR for RP. Administration costs were mainly driven by the cost of pump rented by the healthcare service provider (990 EUR per pump for the 3-month period). No difference was found on indirect costs that included costs for premedication, time lost for injections, and time for getting rid of infusion material (Table 4).

**Table 4:** Analysis of costs per month

	Pump	Rapid push
Direct costs including costs for gammanorm® 165 mg/mL (EUR per month)	1681 ± 628	1320 ± 702
Direct costs excluding costs for gammanorm® 165 mg/mL (EUR per month)	536 ± 176	164 ± 323
Indirect costs (EUR per month)	64 ± 42	69 ± 35

### Safety

Infusions of gammanorm® 165 mg/mL were well tolerated. In total, 17 patients (14 during the pump period and 9 during the RP period) reported a total of 55 adverse events (30 during the pump period and 25 during the RP period). Two adverse events were related to the study drug. One patient experienced general reactions after pump infusion and dropped out from the study before the RP period. One patient reported local pruritus when using RP but did not have a reaction with the pump. He switched back to pump for a few days and then withdrew from the study. All adverse events were of mild or moderate intensity. At least one local reaction was self-reported after 67.2% of RP infusions and 71.8% of pump infusions (*p* = 0.11). Local swelling reactions were less frequent with RP (*p* = 0.003) whereas local pain was less frequent than with pump (p = 0.003; Table 5). More specifically, ten patients experienced at least one infusion associated with local pain during the RP period but three patients accounted for 80 out of 102 (78.4%) infusions with RP associated with local pain. Six patients experienced at least one infusion associated with local pain during the P period but one patient accounted for 11 out of 18 (61.1%) infusions with P associated with local pain. One patient experienced pain during 92% of infusions with P and 88% of infusions with RP.

**Table 5:** Local reactions

	Pump 355 infusions	Rapid push 989 infusions
Redness	141 (39.7%)	386 (39.0%)
Swelling	221 (62.3%)	524 (53.0%)
Induration	86 (24.2%)	226 (22.9%)
Pruritus	6 (1.7%)	11 (1.1%)
Pain	18 (5.1%)	102 (10.3%)
At least one local reaction	255 (71.8%)	665 (67.2%)

## Discussion

This randomized, open-label, crossover study compared the subcutaneous administration of IgG by an infusion pump followed by manual RP, or vice versa in patients who had a long history of SCIg with pump. Our study was based on a rigorous prospective and controlled design in which each patient was exposed to both types of administration in a randomized order. The anticipated benefits of RP were higher flow rates, shorter duration of infusions, and lower volumes per infusion. On the other hand, the higher frequency of injections could be a matter of concern for some patients. As planned by the protocol, Ig exposure was similar during each period. Therefore, periods differed only in the volume of each infusion and frequency of infusions. This ensured that any highlighted difference could not originate from different treatment exposures. On one hand, treatment interference on daily life (PID-LQI factor I) was higher with RP than the one with pump; the non-inferiority hypothesis had to be rejected. On the other hand, no difference was found on other LQI factors (treatment-related problems or therapy setting), global satisfaction, quality of life, and burden of the disease or of IgRT delivery. Moreover, despite the majority of patients preferring the pump administration, 8 patients out of 28 (28.6%) declared to prefer RP. Serum IgG levels resulting from RP infusions were similar to those from pump infusions as previously observed [11]. Overall, the 3-month incidence infection rate was similar during pump and RP periods; although in the latter case, it tended to lower but was associated with the occurrence of one severe infection. The study was not designed to compare efficacy outcomes such as infection rate, especially in patients already receiving IgRT. Interestingly, others have evaluated efficacy data in patients receiving SCIg. Ochs et al. reported an annual infection rate of 4.43 per patient-year in patients receiving SCIg [22]. Here, 3-month incidence rate was estimated to be 1.00 [0.68; 1.47] for pump period and 0.76 [0.49; 1.20] for the RP period, which was relatively in line with previous results. Although data collection during longer periods is warranted, our results supported the efficacy of RP infusions.

As expected, RP infusions were five- to sixfold faster but more than threefold more frequent than pump infusions. Despite not reaching statistical significance, the difference in satisfaction as assessed by TSQM was lower with RP than that with pump. One could not rule out the possibility that higher frequency of infusions may have played a role in these results. It may also be possible that, as a manual procedure, RP required more effort to deliver SCIg. However, validated tools used in this study could not specifically highlight these issues. Patient interviews may be more suited to capture such feedbacks. A dedicated study may be warranted to qualitatively explore them. Furthermore, our patients were accustomed to using pumps for a fairly long time before enrollment. Therefore, switch to RP may have disrupted their well-established routine care. It is possible that new rituals would require a habituation period exceeding 3 months. Unfortunately, the duration of each crossover period was limited to 3 months and we have no data that could suggest that satisfaction would increase with time. Shapiro et al. [11] have reported the results from 104 PID patients who started SCIg and were given the choice of pump or RP. Patients were free to switch to the alternative method at any time during the study. Among these patients with no previous experience with SCIg, 74 initially chose RP and only 9 (12.2%) wished to switch to pump during the study. On the other hand, among 29 patients who started with pump, 13 (44.8%) chose to switch to RP. In contrast to our study which included only adult patients, two thirds of patients in Shapiro’s cohort were less than 18 years old. Maybe most importantly, given that they had no previous experience with pump, RP did not disturb any established routine. In our study, no difference was found on LQI factor II (therapy-related problems) suggesting that no peculiar administration difficulty arose when using RP. As expected, LQI factor III (therapy setting) did not vary since pumps and RP were both used at home. Our results were consistent with a previous preliminary study on patients in the context of a dedicated Patient Support Program aiming at accompanying patients’ move from hospital care to home treatment [23].

No difference on age, gender, and occupation was found between patients who preferred RP rather than pump and those who preferred pump. Half of patients with a preference for RP were living alone. Unfortunately, we did not collect details on occupation which might have suggested that some patients with frequent professional travels would prefer RP or pump.

Infusions were well tolerated independently of the delivery device as suggested by the limited number of patients who took painkillers or other premedication before infusions. Of abdominal infusions with RP, 81.7% were accomplished with a flow rate superior to 35 mL/h. Similar data were reported by Shapiro et al. [11]. Good tolerability of high flow rates has also been demonstrated with SCIg administration by pump [24]. RP allows administrating fairly high volumes in a short time without deteriorating the patient comfort. Local reactions with pump were in general as frequent as those with RP. The rate of infusions associated with local pain was higher with RP. This was not anticipated since patients have been instructed to adapt the flow rate to their own comfort. Painkiller premedication and pain experience during infusion were limited to few patients suggesting that patient component seems to play a marked role.

Home-based SCIg infusions have already been shown to be more cost effective than hospital-based IVIg infusions [25]. Furthermore, Martin et al. demonstrated that home-based RP was less expensive than hospital-based IVIg with a $5736 saving over 3 years [26]. Here, we showed that after exclusion of costs directly related to Ig, monthly costs were 70% less with RP than with pump. It should be highlighted that indirect costs, which are mainly driven by the time spent by the patients for infusion were similar between the two methods. Shorter but more frequent infusions with RP require the same amount of time per month than weekly pump infusions. Finally, an important part of direct costs during the pump period was driven by the costs of the device itself including fixed costs and monthly rental costs. Fixed costs have been amortized over the pump period. Had the study been longer, these costs would have been amortized on the longer time and direct costs would have been decreased. It seems unlikely, however, that administration per pump could be competitive against RP.

Our study has some limitations. Firstly, it has been conducted in only adult patients and satisfaction regarding RP might be different in younger or pediatric patients. Secondly, the sample size was calculated in order to warrant sufficient power for the comparison of LQI factor I. Comparisons of other variables such as TSQM-11 or dimensions of SF36v2 may lack power.

Frequency, dose, route of administration, home or infusion-center administration, and the use of self- or healthcare-professional-administered infusion can be tailored to suit individual patient needs and circumstances [27]. Here, RP proved to be preferred by about one third of the patients and to be more cost-effective than pump infusion. Our results suggest that RP can be a valuable and well-accepted alternative for SC self-administration of gammanorm® 165 mg/mL at home for many PID patients. Since IgRT is a lifelong treatment in PID patients, individualization of treatment is of paramount importance. RP is a new, safe, and easy-to-learn method that complements the physician’s armamentarium when prescribing IgRT.

## Abbreviations

CI: Confidence interval
Ig: Immunoglobulin
IgRT: Immunoglobulin replacement therapy
IV: IgIntravenous immunoglobulin
PID: Primary immunodeficiency
SCIg: Subcutaneous immunoglobulin

## References

[1] Al-Herz W, Bousfiha A, Casanova JL, Chapel H, Conley ME, Cunningham-Rundles C (2011). Primary immunodeficiency diseases: an update on the classification from the international union of immunological societies expert committee for primary immunodeficiency. Front Immunol.

[2] Gardulf A (2007). Immunoglobulin treatment for primary antibody deficiencies: advantages of the subcutaneous route. BioDrugs.

[3] Busse PJ, Razvi S, Cunningham-Rundles C (2002). Efficacy of intravenous immunoglobulin in the prevention of pneumonia in patients with common variable immunodeficiency. J Allergy Clin Immunol.

[4] Gardulf A, Nicolay U (2006). Replacement IgG therapy and self-therapy at home improve the health-related quality of life in patients with primary antibody deficiencies. Curr Opin Allergy Clin Immunol.

[5] Gardulf A, Bjorvell H, Andersen V, Bjorkander J, Ericson D, Froland SS, Gustafson R, Hammarstrom L, Nystrom T, Soeberg B, Smith CIE (1995). Lifelong treatment with gammaglobulin for primary antibody deficiencies: the patients' experiences of subcutaneous self-infusions and home therapy. J Adv Nurs.

[6] Nicolay U, Kiessling P, Berger M, Gupta S, Yel L, Roifman CM, Gardulf A, Eichmann F, Haag S, Massion C, Ochs HD (2006). Health-related quality of life and treatment satisfaction in North American patients with primary immunedeficiency diseases receiving subcutaneous IgG self-infusions at home. J Clin Immunol.

[7] Gardulf A, Nicolay U, Math D, Asensio O, Bernatowska E, Bock A (2004). Children and adults with primary antibody deficiencies gain quality of life by subcutaneous IgG self-infusions at home. J Allergy Clin Immunol.

[8] Abolhassani H, Sadaghiani MS, Aghamohammadi A, Ochs HD, Rezaei N (2012). Home-based subcutaneous immunoglobulin versus hospital-based intravenous immunoglobulin in treatment of primary antibody deficiencies: systematic review and meta analysis. J Clin Immunol.

[9] Shapiro RS (2013). Why I use subcutaneous immunoglobulin (SCIG). J Clin Immunol.

[10] Kobrynski L (2012). Subcutaneous immunoglobulin therapy: a new option for patients with primary immunodeficiency diseases. Biologics.

[11] Shapiro R (2010). Subcutaneous immunoglobulin therapy by rapid push is preferred to infusion by pump: a retrospective analysis. J Clin Immunol.

[12] Shapiro RS (2013). Subcutaneous immunoglobulin: rapid push vs. infusion pump in pediatrics. Pediatr Allergy Immunol.

[13] Gardulf A (2016). Clinical experiences in primary and secondary immunodeficiencies and immune-mediated conditions using gammanorm((R)). Immunotherapy.

[14] Bharmal M, Payne K, Atkinson MJ, Desrosiers MP, Morisky DE, Gemmen E (2009). Validation of an abbreviated Treatment Satisfaction Questionnaire for Medication (TSQM-9) among patients on antihypertensive medications. Health Qual Life Outcomes.

[15] Ware JESK, Kosinski M (2000). SF36 health survey. Manual and interpretation guide.

[16] Buchi S, Buddeberg C, Klaghofer R, Russi EW, Brandli O, Schlosser C (2002). Preliminary validation of PRISM (pictorial representation of illness and self measure)—a brief method to assess suffering. Psychother Psychosom.

[17] Buchi S, Sensky TPRISM (1999). Pictorial representation of illness and self measure. A brief nonverbal measure of illness impact and therapeutic aid in psychosomatic medicine. Psychosomatics.

[18] Nicolay U, Haag S, Eichmann F, Herget S, Spruck D, Gardulf A (2005). Measuring treatment satisfaction in patients with primary immunodeficiency diseases receiving lifelong immunoglobulin replacement therapy. Qual Life Res.

[19] Atkinson MJ, Sinha A, Hass SL, Colman SS, Kumar RN, Brod M, Rowland CR (2004). Validation of a general measure of treatment satisfaction, the treatment satisfaction questionnaire for medication (TSQM), using a national panel study of chronic disease. Health Qual Life Outcomes.

[20] Direction générale de l’offre de soins. Guide pour le suivi de la masse salariale. Ministère chargé de la santé. 2014 [cited 21/03/2017]; Available from: http://social-sante.gouv.fr/IMG/pdf/dgos_guide_suivi_masse_salariale_2014.pdf

[21] Chaput H, Pinel C, Wilner L. Salaires dans le secteur privé et les entreprises publiques - En 2013, le salaire net moyen baisse de 0,3% en euros constants. Insee Première 2015 16/09/2015;1565.

[22] Ochs HD, Gupta S, Kiessling P, Nicolay U, Berger M (2006). Safety and efficacy of self-administered subcutaneous immunoglobulin in patients with primary immunodeficiency diseases. J Clin Immunol.

[23] Reniers A, Heijmans C, Nols N, Rombaut B, Peche R. Subcutaneous administration of gammanorm in immune-deficient patients by rapid push: report of the Belgian experience. J Clin Immunol; 2014: SPRINGER/PLENUM PUBLISHERS 233 SPRING ST, NEW YORK, NY 10013 USA; 2014. p. S507-S.

[24] Hansen S, Gustafson R, Smith CI, Gardulf A (2002). Express subcutaneous IgG infusions: decreased time of delivery with maintained safety. Clin Immunol.

[25] Beaute J, Levy P, Millet V, Debre M, Dudoit Y, Le Mignot L (2009). Economic evaluation of immunoglobulin replacement in patients with primary antibody deficiencies. Clin Exp Immunol.

[26] Martin A, Lavoie L, Goetghebeur M, Schellenberg R (2013). Economic benefits of subcutaneous rapid push versus intravenous immunoglobulin infusion therapy in adult patients with primary immune deficiency. Transfus Med.

[27] Jolles S, Orange JS, Gardulf A, Stein MR, Shapiro R, Borte M, Berger M (2015). Current treatment options with immunoglobulin G for the individualization of care in patients with primary immunodeficiency disease. Clin Exp Immunol.

